# Circulating Tumor Cells Identify Early Recurrence in Patients with Non-Small Cell Lung Cancer Undergoing Radical Resection

**DOI:** 10.1371/journal.pone.0148659

**Published:** 2016-02-25

**Authors:** Clara Bayarri-Lara, Francisco G. Ortega, Antonio Cueto Ladrón de Guevara, Jose L. Puche, Javier Ruiz Zafra, Diego de Miguel-Pérez, Abel Sánchez-Palencia Ramos, Carlos Fernando Giraldo-Ospina, Juan A. Navajas Gómez, Miguel Delgado-Rodriguez, Jose A. Lorente, María Jose Serrano

**Affiliations:** 1 Department of Thoracic Surgery, Virgen de las Nieves University Hospital, Av de las Fuerzas Armadas, 2, 18014, Granada, Spain; 2 GENYO, Centre for Genomics and Oncological Research (Pfizer / University of Granada / Andalusian Regional Government), PTS Granada Av. de la Ilustración, 114–18016, Granada, Spain; 3 Integral Oncology Division, San Cecilio Clinical University Hospital, Av. Dr. Olóriz 16, 18012, Granada, Spain; 4 Division of Preventive Medicine and Public Health, CIBERESP, University of Jaén, Campus de las Lagunillas, Ctra Torrequebradilla s/n, 23071, Jaén, Spain; 5 Laboratory of Genetic Identification, Department of Legal Medicine, University of Granada, Avda. Madrid 11, 18012, Granada, Spain; The Ohio State University, UNITED STATES

## Abstract

**Background:**

Surgery is the treatment of choice for patients with non-small cell lung cancer (NSCLC) stages I-IIIA. However, more than 20% of these patients develop recurrence and die due to their disease. The release of tumor cells into peripheral blood (CTCs) is one of the main causes of recurrence of cancer. The objectives of this study are to identify the prognostic value of the presence and characterization of CTCs in peripheral blood in patients undergoing radical resection for NSCLC.

**Patients and Methods:**

56 patients who underwent radical surgery for previously untreated NSCLC were enrolled in this prospective study. Peripheral blood samples for CTC analysis were obtained before and one month after surgery. In addition CTCs were phenotypically characterized by epidermal growth factor receptor (EGFR) expression.

**Results:**

51.8% of the patients evaluated were positive with the presence of CTCs at baseline. A decrease in the detection rate of CTCs was observed in these patients one month after surgery (32.1%) (p = 0.035). The mean number of CTCs was 3.16 per 10 ml (range 0–84) preoperatively and 0.66 (range 0–3) in postoperative determination. EGFR expression was found in 89.7% of the patients at baseline and in 38.9% patients one month after surgery. The presence of CTCs after surgery was significantly associated with early recurrence (p = 0.018) and a shorter disease free survival (DFS) (p = .008). In multivariate analysis CTC presence after surgery (HR = 5.750, 95% CI: 1.50–21.946, p = 0.010) and N status (HR = 0.296, 95% CI: 0.091–0.961, p = 0.043) were independent prognostic factors for DFS.

**Conclusion:**

CTCs can be detected and characterized in patients undergoing radical resection for non-small cell lung cancer. Their presence might be used to identify patients with increased risk of early recurrence.

## Introduction

Lung cancer represents the leading cause of cancer related death for both men and women, and even in early stages outcomes remain poor [1]. Despite optimal surgical treatment more than 20% of patients designated as early stages by conventional criteria will recur and eventually die of recurrent non-small cell lung cancer (NSCLC) [2,3]. This group of patients with worse than expected prognosis makes it necessary to improve risk stratification with more sensitive prognostic factors. These new prognostic factors must be the result of a better understanding of metastatic process, intimately linked with the detection of CTCs.

In this regard increasing evidence suggests that early relapse in resected NSCLC patients may arise from circulating tumor cells (CTCs) that shed from the primary tumor into the vascular system since the beginning of the malignant process. Detection of these CTCs may identify patients with a high risk of recurrence and could be a more specific indication for adjuvant treatment in these patients [4].

Prognostic value of CTC enumeration has been shown in several types of epithelial tumors and a worse survival has been described in patients with a higher number of CTCs in breast, prostate and colorectal cancer [5–8]. In lung cancer most clinical studies have focused on CTCs detection in metastatic cancer patients as a prognostic factor and as a biomarker of response to treatment [9]. Isolation of CTCs in early stage NSCLC is more challenging and only a few articles can be found suggesting that CTCs numbers can help to predict prognosis [10–13].

Recent findings about CTCs suggest that simply enumeration at a certain moment may not be sufficient [14,15]. It has been proved that CTCs are actually a heterogeneous population and it is known that the genetic and phenotypic characteristics of tumor cells are known to change over time in treated patients. Therefore, not only enumeration but also CTC characterization should be performed at different moments along the follow up. CTC characterization may contribute to identify different subpopulation of CTCs with different possible implications in patient prognosis [14]. Moreover CTC characterization could provide information about genotypic and phenotypic features of a tumour without the need for an invasive biopsy, for instance the detection of epidermal growth factor receptor (EGFR) mutations in NSCLC patients [15]. Nevertheless, the existence of possible discordance between genotypes obtained by tumor biopsy and that determined in CTCs must be taken into account. It has been described in a recent study that relates these discordances with technological differences as well as sampling different tumour cell populations [16].

Epithelial growth factor receptor (EGFR) has been identified in various human tumors, including cancers of breast, ovary, oropharynx, and esophagus, and has predicted poor patient outcomes [17]. Mutations that lead to EGFR overexpression or overactivity have been associated with a number of cancers, including lung cancer. These somatic mutations involving EGFR lead to its constant activation, which produces uncontrolled cell division [18].

Therefore, we are interested in exploring the presence of CTCs and to determine EGFR expression in CTCs in patients with NSCLC. CTC detection and characterization might become a valuable tool to determine prognosis and serve as a real-time tumor biopsy for individually tailored cancer treatment.

In this context, we conducted this study with the following goals: (i) to determine whether CTCs are detectable in patients undergoing radical resection for NSCLC, (ii) to phenotypically characterize these CTCs and (iii) to assess the prognostic significance of CTC detection in these patients in terms of recurrence and disease free survival.

## Patients and Methods

### Study design and patients

Given this background we conducted a prospective longitudinal cohort study of 56 patients with NSCLC who underwent a curative resection at the Department of Thoracic Surgery, Virgen de las Nieves University Hospital at Granada (Spain), between November 2012 and January 2014. Peripheral blood samples were collected before the operation (2–16 hours before) and one month after surgery and were sent to GENyO Centre (Centre for Genomic and Oncological Research located in Granada) for CTC analysis. Control blood samples were drawn from 16 healthy volunteers with no history of malignant disease. Informed consent was obtained from each patient, and the study was approved by the local ethics committee. Median follow up for all patients was 16 months (range 3–23). Clinical outcomes were evaluated in terms of recurrence and disease free survival. Data were collected for age, gender, smoking status, histological subtype, SUV (standardized uptake value), value of positron emission tomography–computed tomography (PET CT), surgical approach, extent of resection, pathological stage, adjuvant treatment, survival, and response.

All 56 patients with NSCLC underwent anatomical pulmonary resection and systematic lymph node dissection with curative intent. Complete resection was defined as demonstrating cancer-free surgical margins, both grossly and histologically. None of these patients received induction chemotherapy or radiotherapy. Another exclusion criteria was either concurrent or prior malignancy in the previous 5 years and death within 30 days of operation. In addition, preoperative evaluation included history, physical examination, and laboratory and radiographic studies including PET CT.

The clinicopathological characteristics of these 56 patients are summarized in Table 1.

**Table 1:** Main demographic, clinical, pathological and treatment related characteristics in NSCLC patients included in the study.

	N	%
**Gender**		
**Male**	50	89,3%
**Female**	6	10,7%
**Age (years)**		
**Mean**	67,4 (45–80)	
**<70**	31	55,4%
**≥70**	25	44,6%
**Smoking status**		
**Exsmoker**	35	62,5%
**Non**	3	5,4%
**Current**	18	32,1%
**PET (max SUV value)**		
**Mean**	13,17 (2,5–25,2)	
**< 7**	11	19,6%
**≥ 7**	45	80,4%
**Hemithorax**		
**Right**	30	53,6%
**Left**	26	46,4%
**Surgical approach**		
**Thoracotomy**	36	64,3%
**VATS**	20	35,7%
**Type of resection**		
**Lobectomy**	44	78,6%
**Pneumonectomy**	8	14,3%
**Segmentectomy**	4	7,1%
**Histology**		
**Adenocarcinoma**	25	44,6%
**Large Cell Carcinoma**	2	3,6%
**Squamous Cell Carcinoma**	29	51,8%
**Pathological stage**		
**IA**	15	26,8%
**IB**	11	19,6%
**IIA**	12	21,4%
**IIB**	10	17,9%
**IIIA**	8	14,3%
**Nodal status**		
**N1**	8	14,3%
**N2**	5	8,9%
**N0**	43	76,8%
**Tumour size (cm)**		
**Median,range**	3,6 (0,8–10)	
**< 3**	23	41,1%
**≥ 3**	33	58,9%
**Adjuvant chemotherapy**		
**No**	35	62,5%
**Yes**	21	37,5%

(N: Number of patients)

Pathologic stage was determined in accordance with the 7th edition of the international tumor-node-metastasis (TNM) system. Histologic subtypes of lung cancer were assigned according to the World Health Organization classification.

Indication for adjuvant treatment was established by the Local Tumor Committee according to ASCO guidelines. In case of adjuvant treatment it was initiated after the second blood extraction.

The follow-up schedule was the usually performed for resected NSCLC and it consisted of a first clinical visit one month after surgery, then one every 3 months in the first year after resection and finally one every 6 months from the second to the fifth year. At least one chest CT scan was performed every 6 months and a PET-CT study per year.

Local recurrence was defined as that occurring at the following sites: ipsilateral lung, bronchial stump or staple line, and regional lymph node (subcarinal, periesophageal, ipsilateral or contralateral mediastinum, supraclavicular, or hiliar lymph nodes).

Distant recurrence included metastases in the contralateral lung, liver, adrenal glands, brain, bone, or other locations. Patterns of recurrence were determined by clinical assessment, radiographic test and supplementary data from bronchoscopy, biopsy, and PET.

This study was approved by the ethical Committee of the Hospital. Written informed consent was obtained from all the cancer patients and healthy volunteers in this study.

### Statistical Analysis

Patients were considered CTC positive if at least one CTC was isolated. CTCs were assessed both as continuous variable and as binary variable (presence/absence). The relationships between CTCs and other variables were ascertained by a t-test and ANOVA (comparison of means) when CTC were continuous and Fisher's exact test when CTC were considered as a binary variable. To compare CTCs measured at two different times the Wilcoxon signed-rank test was used. The influence of CTC on disease-free survival was measured by univariate methods (Kaplan-Meier) and multivariate procedures (Cox regression). In the selection of variables to be included in multivariate Cox regression, the criterion of more than a 10% change in the CTC coefficient estimate was applied [19].

### Enumeration and Characterization of CTCs by CK and EGFR expression

A total of 20 ml of blood were collected from each donor in two Cell Save Preservatives blood collection Tubes (Veridex, LLC, Johnson & Johnson Company) CTC detection was performed by positive immunomagnetic selection. Samples were processed according to the protocol we have previously established [8] (S1 Text).

**Fig 1:**
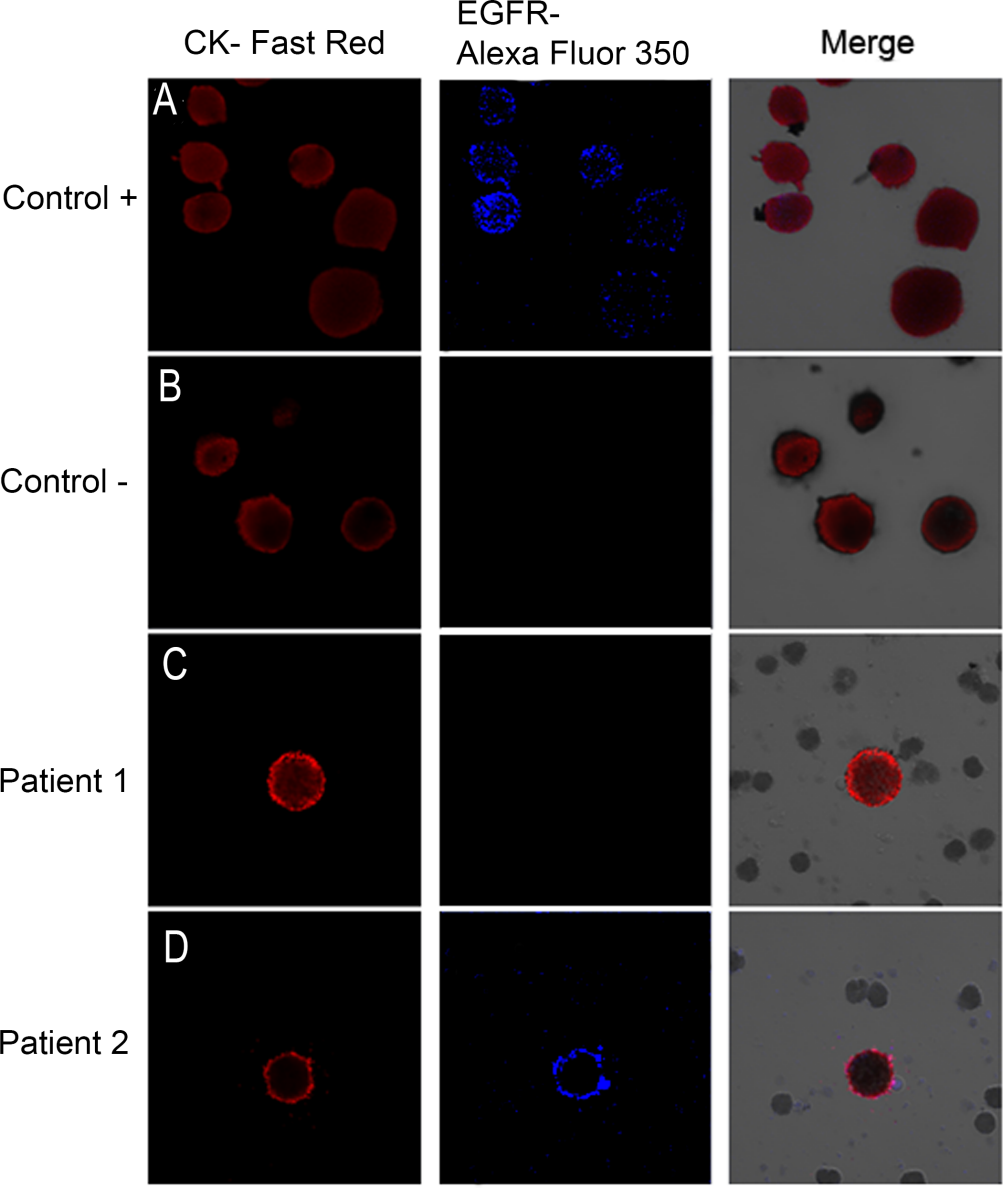
Image galleries after isolation, cytomorphological analysis, and detection of cytokeratin-positive (CK+) cells (red staining) and epidermal growth factor receptor (EGFR). A) T1975 cell tumor line. was used as positive control for EGFR expression B) A control in which the slides are incubated with antibody diluent, without the primary antibody was included. (C, D) Expression of different markers in patients with non-small lung cancer through combination of stained CK+ cells (red) with EGFR (blue). EGFR-specific immunofluorescence (IF) of circulating tumor cells (CTCs) was determined with Alexa 355.

Briefly, the samples were processed by density gradient centrifugation (Histopaque 1119; Sigma-Aldrich, St. Louis, MO, USA). For CTC enrichment, we used the Carcinoma Cell Enrichment and Detection kit with MACS technology (Miltenyi Biotec, Bergisch Gladbach, Germany). CTCs were identified by immunocytochemical methods and visualized under a direct light microscope to perform the combined cytomorphological and immunophenotypic assessment. CK-positive and EGFR-positive cells were identified by immunohistochemistry (IHC) and signals were detected by chromogenic and fluorescent detection, respectively [20] Epithelial tumor cells were identified and enumerated based on their red staining for CK-positive cells and blue staining for EGFR-positive cells (Fig 1).

Specific staining can easily be distinguished due to the differential intracellular distribution of the examined molecules and the combination of direct and indirect IF in order to evaluate CK+/EGFR+.

## Results

### CTCs detection and correlation with clinicopathological characteristics

CTCs were detected in peripheral blood of 51.8% of patients at baseline (CTC1) (29 of 56 patients) Interestingly, we observed that the detection rate of CTCs in these patients was significantly lower one month after surgery (CTC2) (18 patients, 32.1%) p = 0.034, yielded from the Wilcoxon signed-rank test. The mean number of CTCs was 3.16 per 10 ml (median 1; range 0–84) preoperatively and it decreased to 0.66 (median 0; range 0–3) after the operation (S1 Table) (p = 0.051). Dynamic changes in CTCs number were also determined observing that most patients with CTCs at baseline presented a decrease in CTCs count (25 out of 29) after surgery. On the other hand only 11 patients showed an increase.

Correlation between CTCs presence and patient characteristics is summarized in Table 2.

**Table 2:** Correlation between CTCs status before (CTC1) and after surgery (CTC2) and clinicopathological characteristics.

		CTC1			CTC2		
		N(%) +	N(%) -	p	N(%) +	N(%) -	p
**Age (years)**	**< 70**	13 (41.9%)	18 (58.1%)	0.116	11 (35.5%)	20 (64.5%)	0.58
	**≥ 70**	16 (64%)	9 (36%)		7 (28%)	18 (72%)	
**Gender**	**Male**	27 (54%)	23 (46%)	0.301	17 (34%)	33 (66%)	0.363
	**Female**	2 (33.3%)	4 (66.7%)		1 (16.7%)	5 (83.3%)	
**p TNM Stage**	**I**	13 (50%)	13 (50%)	0.508	7 (26.9%)	19 (73.1%)	0.313
	**II-III**	16 (53.3%)	14 (46.7%)		11 (36.7%)	19 (63.3%)	
**Nodal status**	**N0**	21 (48.8%)	22 (51.2%)	0.315	14 (32.6%)	29 (67.4%)	0.594
	**N1-N2**	8 (61.5%)	5 (38.5%)		4 (30.8%)	9 (69.2%)	
**Tumor size**	**< 3**	13 (56.5%)	10 (43.5%)	0.375	7 (30.4%)	16 (69.6%)	0.527
**(cm)**	**≥ 3**	16 (48.5%)	17 (51.5%)		11 (33.3%)	22 (66.7%)	
**PET**	**< 7**	7 (63.6%)	4 (36.4%)	0.380	4 (36.4%)	7 (63.6%)	0.738
**(max SUV value)**							
	**≥ 7**	22 (48.9%)	23 (51.1%)		14 (31.1%)	31 (68.9%)	
**Histology**	**Adenocarcinoma**	11 (44%)	14 (56%)	0.218	8 (32%)	17 (68%)	0.606
	**Other**	18 (58.1%)	13 (41.9%)		10 (32.3%)	21 (67.7%)	
**Surgical approach**	**Thoracotomy**	18 (50%)	18 (50%)	0.469	12 (33.3%)	24 (66.7%)	0.521
	**VATS**	11 (55%)	9 (45%)		6 (30%)	14 (70%)	
**Type of ressection**	**Pneumonectomy**	4 (50%)	4 (50%)	0.605	0 (0%)	8 (100%)	**0.034**
	**Other**	25 (52.1%)	23 (47.9%)		18 (37.5%)	30 (62.5%)	

(N: Number of patients)

In the univariate analysis only the type of resection showed a significant correlation with CTC presence after surgery (P = 0.034). 4/4 patients underwent pneumonectomy resection changed from CTC1+ to CTC2-, while 7 of 25 who had another type of resection changed to CTC2-. CTC count in the second detection was correlated with max SUV of PET scan (β = 0.05 95%; 95% CI 0.001–0.106, p = 0.046). In the multivariate analysis there was no significant correlation between CTC presence or number and any clinicopathological characteristic analysed.

### Characterization of EGFR in CTCs

The CTCs were also analyzed for epidermal growth factor receptor (EGFR) expression, EGFR expression was found in 26 patients at baseline and in 7 patients one month later. For CTC positive cases EGFR expression rate was 89.7% before and 39% after surgery. There was no significant correlation between CTCs EGFR+ status and clinico-pathological characteristics.

### Prognostic significance of CTCs detection

During the follow up 16 patients showed evidence of cancer recurrence (28.6%). Median time to recurrence was 8 months, ranging between 2 and 17 months after surgery. Pattern of recurrence was: distant 7, locoregional 4 and both 5. For the entire cohort 1-year DFS rate was 76.6%. Association between recurrence and most important prognostic factors was analyzed (Table 3). Only CTC presence after surgery was significantly associated with early recurrence, 50% of patients with detectable CTCs after surgery (CTC2+) developed a recurrence compared to 18.4% for CTC2 negative patients (p = 0.018). Regarding nodal extension, N1-N2 positive patients presented a higher percentage of recurrence than N0 patientes althought the difference was not significant (p = 0.161).

**Table 3:** Recurrence rate according to patient characteristics and CTCs status.

		Recurrence				p
		yes		no		
**Age (years)**						
** **	**< 70**	8	25.8%	23	74.2%	0.767
** **	**≥ 70**	8	32%	17	68%	
**Histological type**						
** **	**Adenocarcinoma**	7	28%	18	72%	0.932
** **	**Other**	9	29%	22	71%	
**PET (maxSUV value)**						
** **	**< 7**	5	45.5%	6	54.5%	0.263
** **	**≥ 7**	11	24.4%	34	75.6%	
**Pathological stage**						
** **	**I**	7	26.9%	19	73.1%	0.518
** **	**II + III**	9	30%	21	70%	
**Tumoral size (cm)**						
** **	**< 3**	5	21.7%	18	78.3%	0,262
** **	**≥ 3**	11	33.3%	22	66.7%	
**N status**						
** **	**N0**	10	23.3%	33	76.7%	0.161
** **	**N1-N2**	6	46.2%	7	53.8%	
**Surgical approach**						
** **	**Thoracotomy**	10	27.8%	26	72.2%	0.547
** **	**VATS**	6	30%	14	70%	
**Type of resection**						
** **	**Other**	13	27.1%	35	72.9%	0.676
** **	**Pneumonectomy**	3	37.5%	5	62.5%	
**Adjuvant chemotherapy**						
** **	**Yes**	5	23.8%	16	76.2%	0.384
** **	**No**	11	31.4%	24	68.6%	
**CTC 1**						
** **	**Yes**	10	34.5%	19	65.5%	0.237
** **	**No**	6	22.2%	21	77.8%	
**CTC 2**						
** **	**Yes**	9	50%	9	50%	**0.018**
** **	**No**	7	18.4%	31	81.6%	
**EGFR+ CTC1**						
** **	**Yes**	9	34.6%	17	65.4%	0.262
** **	**No**	7	23.3%	23	76.7%	
**EGFR+ CTC 2**						
** **	**Yes**	2	28.6%	5	71.4%	0.655
** **	**No**	14	28.6%	35	71.4%	

Considering only CTCs positive patients, EGFR expression in CTCs didn’t show a significant correlation with tumor recurrence (Table 4) In the second detection 28.6% of EGFR+CTC2+ patients presented a recurrence compared to 63.4% of EGFR-CTC2+ patients but the difference was not siginificant (p = 0.335) (Table 4)

**Table 4:** Recurrence according EGFR expression for CTC positive patients. EGFR1 = EGFR expression in CTCs before surgery. EGFR2 = EGFR expression in CTCs after surgery.

	Recurrence				n	p
	Yes		no			
**EGFR 1**						
**+**	9	34.6%	17	65.4%	26	1
**-**	1	33.3%	2	66.7%	3	
**EGFR 2**						
**+**	2	28.6%	5	71.4%	7	0.335
**-**	7	63.6%	4	36.4%	11	

n = number of patients.

**Fig 2:**
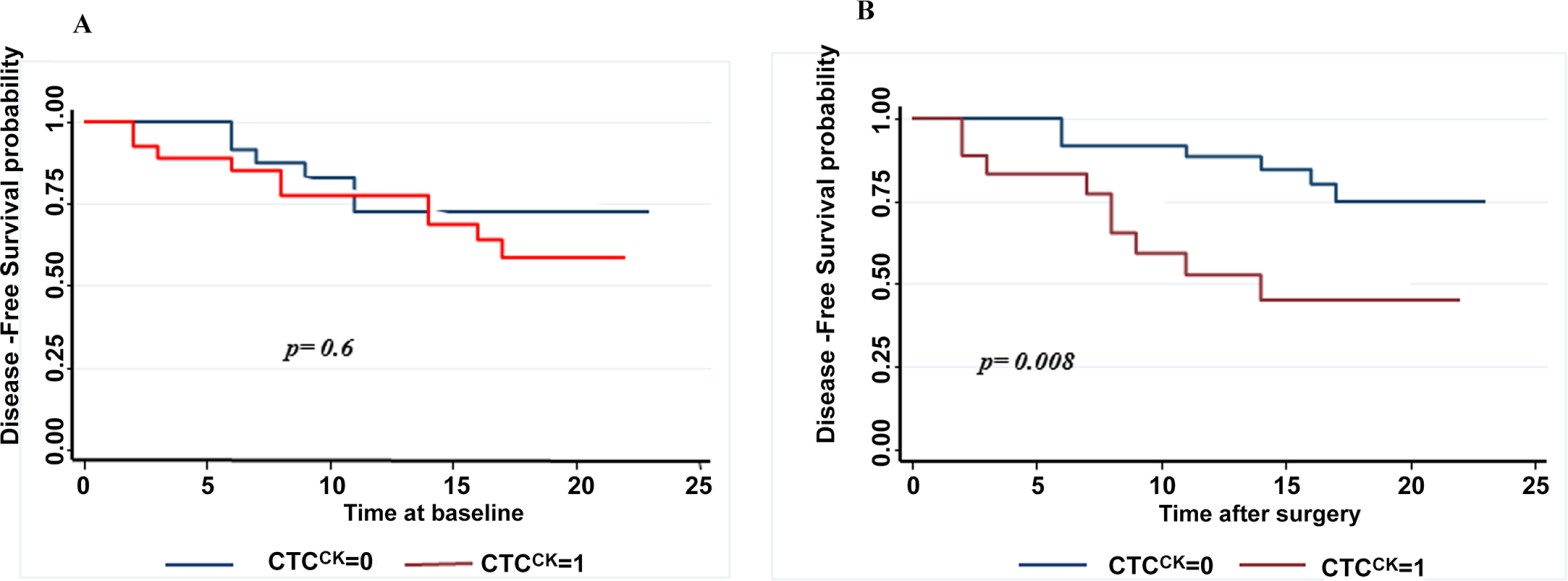
Kaplan-Meier curves of disease free survival according to CTC status before (A) and one month after surgery (B). CTC1: CTC presence before surgery. CTC2: CTC presence after surgery.

In addition CTC presence after surgery (CTC2) was associated with a shorter DFS, with one year DFS rate of 51% compared to 87.7% for CTC negative group after surgery (log rank test p = 0.008) (Fig 2). Although the mean number of CTC after surgery was greater for patients with recurrence, the difference was not significant (1 cell per 10 ml vs 0.5 per 10 ml, p = 0.13).

The detection of CTCs after surgery was significantly correlated with a shorter DFS in patients undergoing radical resection for Non-Small-Cell Lung Cancer.

Prognostic factors for disease-free survival were identified by the univariate Cox proportional hazards regression analysis (Table 5). Factors associated with DFS that had a p ≤ 0.1 were CTC presence after surgery (CTC2) (p = 0.014) and N status (p = 0.07)

**Table 5:** Univariate analysis of risk factors for disease-free survival (DFS).

DFS	Univariate Analysis		
PROGNOSTIC FACTORS	HR	95% IC	p
**CTC 1 +**	1.34	0.48–3.71	0.574
**CTC 2 +**	3.481	1.29–9.38	**0.014**
**EGFR CTC 1 +**	1.332	0.49–3.59	0.571
**EGFR CTC 2 +**	1.027	0.23–4.53	0.971
**Histological type**			
**Ohter**	1.07	0.39–2.87	0.89
**Adenocarcinoma**			
**PET (max SUV value)**			
**<7**			
**≥7**	0.535	0.19–1.54	0.247
**Tumor size (cm)**			
**≥3**	1.85	0.64–5.32	0.256
**<3**			
**N status**			
**N1-N2**	2.54	0.92–7.04	0.07
**N0**			
**Surgical approach**			
**VATS**	1.21	0.44–3.34	0.712
**Thoractomy**			
**Type of resection**			
**Pneumonectomy**	1.18	0.34–4.15	0.794
**Other**			
**Adjuvant chemotherapy**			
**Yes**	0.63	0.22–1.82	0.392
**No**			

HR = Hazard ratio; CI = confidential interval; CTC = circulating tumor cells

The multivariate forward stepwise Cox proportional hazards regression analysis, revealed that CTC presence after surgery (HR,5.75; 95% CI 1.50–21.95; p = 0.01) and N status (HR,3.38; 95% CI 1.04–10.9; p = 0.043) were independent prognostic factor for DFS (Table 6).

**Table 6:** Multivariate analysis of risk factors for disease-free survival (DFS).

Multivariate Analysis	DFS			
PROGNOSTIC FACTORS		HR	95% CI	p
**CTC 2 +**		5.750	1.50–21.95	0.010
**Tumor size (cm)**				
**≥3**		3.10	0.72–13.42	0.129
**<3**				
**N status**				
**N1-N2**		3.38	1.04–10.99	0.043
**N0**				
**Surgical approach**				
**VATS**		4.11	0.81–20.85	0.088
**Thoractomy**				
**Type of resection**				
**Pneumonectomy**		3.92	0.61–25.01	0.149
**Lobectomy/segmentectomy**				
**Adjuvant chemotherapy**				
**Yes**		0.44	0.10–1.82	0.256
**No**				

HR = Hazard ratio; CI = confidential interval; CTC = circulating tumor cells

## Discussion

The clinical value of CTCs detection in patients´ blood is becoming increasingly important as cancer biomarker research [14]. The main goal of CTC detection in early stages NSCLC is to identify patients with a high risk of recurrence after surgery in order to adopt the best strategy for treatment and follow up.

In this prospective study we found that CTC presence after surgery is associated with early recurrence and a shorter disease free survival in patients with surgical treatment of NSCLC

In our analysis, which enrolled 56 patients who had undergone radical surgery for previously untreated NSCLC, detection rate was significantly lower one month after surgery (32.14% vs. 51.79% before surgery, p = 0.034).

There are only a few studies that evaluate the prognostic significance of CTCs detection in resected NSCLC and even less that perform a second detection after surgery [10–13]. In a study by Hofman et al. [10] including I-IV stages resectable patients, the detection rate is similar to ours (49%). They perform only one presurgery CTCs detection employing a technique based on isolation by size (ISET technique), and they found that a CTC count of 50 cells or more is correlated with a worse overall and disease free survival. In another small scale study by Rolle et al. [11] 30 patients with resectable NSCLC were examined using the MAINTRAC technique, based on cytometric assay. CTCs were determined 2 weeks and 5 months after surgery. The CTCs count was compared to prognosis and patients with continuously increasing in median CTC-count post-operatively were shown to be at a higher risk of relapse.

Only two more prospective studies [12,13] using CellSearch system to detect CTCs, have reported so far the clinical impact of CTCs detection in peripheral blood before and after surgery. However, both failed to demonstrate any difference in either survival or recurrence. Both of them analyzed CTCs presence in blood immediately after surgery. Sawabata´s group [13] observed that CTCs disappeared ten days after operations. In fact, it is well known that during surgery a large number of CTCs can be shed into the blood stream but it has not been demonstrated that these cells are involved in the development of any future metastases. A number of them are apoptotic and many of them will be eliminated by the immune system [14]. That is why we think that for a real assessment of CTC presence after surgery the second detection must be performed at least 3 weeks after the operation.

Indeed we found a significant decrease in CTCs detection rate one month after surgery: CTCs were not detected in peripheral blood in almost 70% of patients in the second detection, and 25 out of 29 patients with CTCs presence at baseline showed a decrease in CTC count. This finding can be one of the reasons that explains why surgery remains the best treatment option in early stages.

The presence of CTCs one month after radical resection might indicate the presence of another source of CTCs not removed by surgery, such as those undetectable by conventional methods.

In accordance with that, in our study patients with detectable CTCs in peripheral blood one month after surgery presented a worse prognosis. Presence of CTCs after surgery (CTC2) was significantly associated with recurrence and DFS, so that CTC2 positive patients presented a higher risk of recurrence (p = 0.018) and a shorter DFS. The one year DFS rate was 51% in CTC2 positive patients compared to 87% for CTC2 negative patients (log rank test p = 0.008). CTC level was higher in patients with recurrence but the difference was not siginificant (mean number of 1 cell per 10 ml for patients with recurrence vs 0.5 per 10 ml for patients who did not develop a recurrence, p = 0.13).The mere presence of at least one CTCs after surgery (CTC2+) was significantly associated with patient prognosis in our analisys. Our results reflect that the presence of post-surgery CTCs (HR,5.75; 95% CI 1.50–21.95; p = 0.01) and nodal status (HR,3.38; 95% CI 1.04–10.9; p = 0.043) are good independent prognostic factors for DFS in multivariate analysis.

CTCs count in post-surgical samples showed an association with the max SUV of PET scan (p = .046). This is the first time that this association has been analysed. Max Suv value of PET scan has been associated with a worse prognoses and it could be related to a higher risk of postoperative CTCs presence.

Besides detection, CTCs characterization could be important to assess prognosis and the type of adjuvant treatment or to determine the best option of treatment for recurrent disease. EGFR expression was detected in 89.7% and 38.9% of CTCs isolated from blood samples taken before and after surgery, respectively. Regarding the prognostic siginificance of EGFR expression we found that 28.6% of patients with EGFR+CTC2+ experienced a recurrence compared to 63.6% for EGFR-CTC2+, but the difference was not significant. In our study CTCs detection after surgery was associated with worse prognosis but EGFR characterization was not. Probably a large-scale study may determine the EGFR expression influence in these patients prognosis.

On the other hand, it is worth stressing that current methodologies allow us to detect only the subpopulation of CTCs positive for epithelial markers, but not subpopulations positive for expression of mesenchymal markers and with a semi mesenchymal or mesenchymal phenotype. These phenotypes have been correlated with bad prognosis in different type of tumors [21] and therefore they should be analysed in future studies in order to achieve a better understanding of the prognostic role of CTCs in lung cancer.

To our knowledge, this study is the first large data set to validate CTCs as a prognostic marker performing postsurgery detection and to assess not only the risk of early recurrence but also to determine CTCs as predictive markers through their characterization.

In conclusion, our results show that CTCs can be detected in the blood of patients undergoing radical surgery for NSCLC. CTC detection after surgery could be an important prognostic marker contributing to risk stratification by identifying patients with a higher risk of early recurrence. Further studies are warranted to validate these results.

## Supporting Information

S1 Table CTCs recovery rate in lung cancer patients. (DOCX)

S1 Text Detection and characterization of CTCs isolated by immunomagnetic positive selection. (DOCX)

## References

[1] Siegel R, Ma J, Zou Z, Jemal A. Cancer statistics. CA Cancer J Clin. 2014;64(1): 9–29. 10.3322/caac.21208 24399786

[2] Kelsey CR, Marks LB, Hollis D, Hubbs JL, Ready NE, D'Amico TA, et al Local recurrence after surgery for early stage lung cancer: an 11-year experience with 975 patients. Cancer. 2009;115(22): 5218–27. 10.1002/cncr.24625 19672942

[3] Song IH, Yeom SW, Heo S, Choi WS, Yang HC, Jheon S, et al Prognostic factors for post-recurrence survival in patients with completely resected Stage I non-small-cell lung cancer. Eur J Cardiothorac Surg. 2014;45(2): 262–7. 10.1093/ejcts/ezt333 23811122

[4] O'Flaherty JD, Gray S, Richard D, Fennell D, O'Leary JJ, Blackhall FH, et al Circulating tumour cells, their role in metastasis and their clinical utility in lung cancer. Lung Cancer. 2012;76(1): 19–25. 10.1016/j.lungcan.2011.10.018 22209049

[5] Iinuma H, Watanabe T, Mimori K, Adachi M, Hayashi N, Tamura J, et al Clinical significance of circulating tumor cells, including cancer stem-like cells, in peripheral blood for recurrence and prognosis in patients with Dukes’ stage B and C colorectal cancer. J Clin Oncol. 2011;29(12): 1547–55. 10.1200/JCO.2010.30.5151 21422427

[6] Serrano MJ, Nadal R, Lorente JA, Salido M, Rodríguez R, Rodríguez M, et al Circulating cancer cells in division in an early breast cancer patient. Ann Oncol. 2011;22(9): 2150–1. 10.1093/annonc/mdr322 21743104

[7] Nadal R, Fernandez A, Sanchez-Rovira P, Salido M, Rodríguez M, García-Puche JL. Biomarkers characterization of circulating tumour cells in breast cancer patients. Breast Cancer Res. 2012;14(3): R71 22554015 10.1186/bcr3180 PMC3446333

[8] Serrano MJ, Sánchez-Rovira P, Delgado-Rodriguez M, Gaforio JJ. Detection of circulating tumor cells in the context of treatment: prognostic value in breast cancer. Cancer Biol Ther. 2009;8(8): 671–5. 19242121 10.4161/cbt.8.8.7834

[9] Krebs MG, Sloane R, Priest L, Lancashire L, Hou JM, Greystoke A, et al Evaluation and prognostic significance of circulating tumor cells in patients with non-small-cell lung cancer. J Clin Oncol. 2011;29(12): 1556–63. 10.1200/JCO.2010.28.7045 21422424

[10] Hofman V, Bonnetaud C, Ilie MI, Vielh P, Vignaud JM, Fléjou JF, et al Preoperative circulating tumor cell detection using the isolation by size of epithelial tumor cell method for patients with lung cancer is a new prognostic biomarker. Clin Cancer Res. 2011;17(4): 827–35. 10.1158/1078-0432.CCR-10-0445 21098695

[11] Rolle A, Günzel R, Pachmann U, Willen B, Höffken K, Pachmann K. Increase in number of circulating disseminated epithelial cells after surgery for non-small cell lung cancer monitored by MAINTRAC(R) is a predictor for relapse: A preliminary report. World J Surg Oncol. 2005;3(1): 18 15801980 10.1186/1477-7819-3-18 PMC1087511

[12] Okumura Y, Tanaka F, Yoneda K, Hashimoto M, Takuwa T, Kondo N, et al Circulating tumor cells in pulmonary venous blood of primary lung cancer patients. Ann Thorac Surg. 2009;87(6): 1669–75. 10.1016/j.athoracsur.2009.03.073 19463575

[13] Sawabata N, Okumura M, Utsumi T, Inoue M, Shiono H, Minami M, et al Circulating tumor cells in peripheral blood caused by surgical manipulation of non-small-cell lung cancer: pilot study using an immunocytology method. Gen Thorac Cardiovasc Surg. 2007;55(5): 189–92. 17554991 10.1007/s11748-007-0101-2

[14] Hofman V, Ilie M, Long E, Guibert N, Selva E, Washetine K, et al Detection of circulating tumor cells from lung cancer patients in the era of targeted therapy: promises, drawbacks and pitfalls. Curr Mol Med. 2014;14(4): 440–56. 24730524 10.2174/1566524014666140414205455

[15] Maheswaran S, Sequist LV, Nagrath S, Ulkus L, Brannigan B, Collura CV, et al Detection of mutations in EGFR in circulating lung-cancer cells. N Engl J Med. 2008;359(4): 366–77. 10.1056/NEJMoa0800668 18596266 PMC3551471

[16] Sundaresan TK, Sequist LV, Heymach JV, Riely GJ, Janne PA, Koch WH, et al Detection of T790M, the acquired resistance EGFR mutation, by tumor biopsy versus noninvasive blood-based analyses. Clin Cancer Res. 2015; In press. 10.1158/1078-0432.CCR-15-1031 PMC4775471 26446944

[17] Traynor AM, Weigel TL, Oettel KR. Nuclear EGFR protein expression predicts poor survival in early stage non-small cell lung cancer. Lung Cancer. 2013;81(1): 138–41. 10.1016/j.lungcan.2013.03.020 23628526 PMC3679338

[18] Han W, Lo H-W. Landscape of EGFR signaling network in human cancers: Biology and therapeutic response in relation to receptor subcellular locations. Cancer Lett. 2012;318: 124–134. 10.1016/j.canlet.2012.01.011 22261334 PMC3304012

[19] Maldonado G, Greenland S. Simulation study of confounder-selection strategies. Am J Epidemiol. 1993;138(11): 923–36. 8256780 10.1093/oxfordjournals.aje.a116813

[20] Nadal R, Ortega FG, Salido M, Lorente JA, Rodríguez-Rivera M, Delgado-Rodríguez M, et al CD133 expression in circulating tumor cells from breast cancer patients: potential role in resistance to chemotherapy. Int J Cancer. 2013;133(10): 2398–407. 10.1002/ijc.28263 23661576

[21] Serrano MJ, Ortega FG, Alvarez-Cubero MJ, Nadal R, Sanchez-Rovira P, Salido M, et al EMT and EGFR in CTCs cytokeratin negative non-metastatic breast cancer. Oncotarget. 2014;5(17): 7486–97. 25277187 10.18632/oncotarget.2217 PMC4202138

