# Supplementary material for: Circulating Tumor Cells Identify Early Recurrence in Patients with Non-Small Cell Lung Cancer Undergoing Radical Resection
Source: PLoS One. 2016 Feb 25;11(2):e0148659. doi: 10.1371/journal.pone.0148659 (PMC4767413; doi:10.1371/journal.pone.0148659)
Supplement: S1 Table — (DOCX) [file pone.0148659.s001.docx]

|  | **mean** | **median** | **range** |
| --- | --- | --- | --- |
| CTC1 | 3.16 | 1 | 0-84 |
| CTC2 | 0.66 | 0 | 0-3 |

**CTC count per 10 ml. for all patients (n=56)**

|  | **mean** | **median** | **range** |
| --- | --- | --- | --- |
| **CTC1** | 6.1 | 3 | 1-84 |
| **CTC2** | 2.1 | 2 | 1-3 |

**CTC count per 10 ml. for CTC + patients**

**S1 Table:** CTCs recovery rate in lung cancer patients
