# Supplementary material for: Circulating Tumor Cells Identify Early Recurrence in Patients with Non-Small Cell Lung Cancer Undergoing Radical Resection
Source: PLoS One. 2016 Feb 25;11(2):e0148659. doi: 10.1371/journal.pone.0148659 (PMC4767413; doi:10.1371/journal.pone.0148659)
Supplement: S1 Text — (DOCX) [file pone.0148659.s002.docx]

**SUPPLEMENTARY**

**Detection and characterization of CTCs isolated by immunomagnetic positive selection**

1. **Blood samples**

After informed consent of patients, peripheral venous blood samples (20 ml) were collected in CellSave Preservatives Tubes (Veridex, LLC, Johnson & Johnson Company) and processed within 72 hr.

The study was reviewed and approved by the Hospital Ethics Committee. This way, blood samples from NSCLC patients were extracted before surgery and one month after it. As negative controls, 16 blood samples from healthy volunteers without evidence of an epithelial malignancy were examined. Positive controls were obtained using 10 ml of blood from healthy volunteers spiked with T1975 human lung cancer line cells and processed separately from patient samples, to avoid contamination.

1. **Density gradient separation**

According to Sigma procedure, a gradient was formed by layering 5 ml of HISTOPAQUE- 1119 (Sigma) in conical tubes. Blood samples were carefully layered onto the HISTOPAQUE-1119 medium. Tubes were then centrifuged at 700*g* for 30 min. The GC and MNC fraction was found at the 1119 interphase. Then, cell fractions were mixed and washed in 50 ml PBS.

1. **Magnetic labeling**

Then, cells were magnetically labeled according to the manufacturer protocols (Miltenyi Biotec, Bergisch Gladbach, Germany). Cytokeratin-expressing cells from peripheral blood of breast cancer patients were enriched and detected using the Carcinoma Cell Enrichment and Detection Kit, Immunocytochemistry (Miltenyi Biotec). According to the manufacturer, cells were diluted in 35ml of 1X MACS Dilution buffer, then they were permeabilized by adding 5ml of MACS CellPerm Solution, incubating for 5 min at 20–25°C, and finally fixed upon addition of 5ml of MACS CellFix Solution with another incubation for 30 min at 20– 25°C. Cells were then washed twice in 1X MACS CellStain Solution and resuspended in 600 µl of the same solution. To block Fc receptors, 200 µl of FcR Blocking Reagent were added, and tumor cells were magnetically labeled by adding 200 µl of MACS Cytokeratin MicroBeads (colloidal super-paramagnetic MicroBeads conjugated to a monoclonal anti-cytokeratin 7/8 antibody; Clone: CAM5.2) and incubating for 45 min at 20–25°C. After incubation, cells were stained with 100µl of anti-cytokeratin conjugated to fluorescein isothiocyanate (FITC) for 10 min in the dark at 20–25°C. After washing, cells were resuspended in a final volume of 500 µl CellStain Solution and stained with 10 µl of anti-FITC conjugated to alkaline phosphatase for 10 min in the dark at 20–25°C.

1. **Magnetic cell separation**

For magnetic enrichment of epithelial tumor cells, the magnetically labeled cell suspension was applied to a prefilled positive separation column (MiniMACS separation columns, type MS) in the magnetic field of a MiniMACS platform (Miltenyi Biotec).

Cells were passed through the column and washed three times with 500 µl 1X MACS Dilution Buffer, so negative (non-magnetic) cell population was washed out of the column. Afterward, the column was removed from the gradient magnetic field and the retained cells were collected (magnetic-positive cell population) as elute using the plunger after a dilution buffer washing step with a total volume of 1 ml.

1. **Sample preparation and detection of epithelial tumor cells by immunocytochemistry**

The magnetically enriched cell fractions were spun down onto polylysine-coated glass slides (Sigma) in a cytocentrifuge (Hettich, Tuttlingen, Germany) at 1,500 rpm for 10 min and the slides were air dried overnight at room temperature. Later, the slides were washed in 1X PBS and cytokeratin-expressing cells were revealed by incubation with freshly prepared Fast Red TR/Naphthol AS-MX substrate solution for 15 minutes in humidity chamber at room temperature. CTCs were identified by immunocytochemical methods and visualized under a direct light microscope to perform the combined cytomorphologic and immunophenotypic assessment. The cytomorphological criteria proposed by Meng et al. [1] (for example, high nuclear/cytoplasmic ratio, larger cells than white blood cells) were used to characterize a CK positive cell as a CTC.

Positive slides for CK-positive cells were then stained with EGFR rabbit anti-human primary antibodies and afterward, with monoclonal anti-human EGFR (Dako) diluted 1:25, followed by incubation with Alexa flour 355 (Molecular Probes. Invitrogen). Epithelial tumour cells were identified and enumerated based on their red staining for CK-positive cells and blue staining for EGFR-positive cells. Specific staining can easily be distinguished because of the differential intracellular distribution of the examined molecules and the combination of direct and indirect IF in order to evaluate CK+/ EGFR .

References

1. Meng S, Tripathy D, Frenkel EP, Shete S, et al : Circulating tumor cells in patients with breast cancer dormancy. *Clin Cancer Res*. 10:8152-8162, 2004
